# TV-NARX and Coiflets WPT based time-frequency Granger causality with application to corticomuscular coupling in hand-grasping

**DOI:** 10.3389/fnins.2022.1014495

**Published:** 2022-09-29

**Authors:** Feifei Zhu, Yurong Li, Zhengyi Shi, Wuxiang Shi

**Affiliations:** ^1^College of Electrical Engineering and Automation, Fuzhou University, Fuzhou, China; ^2^Fujian Provincial Key Laboratory of Medical Instrument and Pharmaceutical Technology, Fuzhou University, Fuzhou, China

**Keywords:** TV-NARX, Granger causality, wavelet packet transformation, corticomuscular coupling, hand-grasping, EEG, EMG

## Abstract

The study of the synchronous characteristics and functional connections between the functional cortex and muscles of hand-grasping movements is important in basic research, clinical disease diagnosis and rehabilitation evaluation. The electroencephalogram (EEG) and electromyographic signal (EMG) signals of 15 healthy participants were used to analyze the corticomuscular coupling under grasping movements by holding three different objects, namely, card, ball, and cup by using the time-frequency Granger causality method based on time-varying nonlinear autoregressive with exogenous input (TV-NARX) model and Coiflets wavelet packet transform. The results show that there is a bidirectional coupling between cortex and muscles under grasping movement, and it is mainly reflected in the beta and gamma frequency bands, in which there is a statistically significant difference (*p* < 0.05) among the different grasping actions during the movement execution period in the beta frequency band, and a statistically significant difference (*p* < 0.1) among the different grasping actions during the movement preparation period in the gamma frequency band. The results show that the proposed method can effectively characterize the EEG-EMG synchronization features and functional connections in different frequency bands during the movement preparation and execution phases in the time-frequency domain, and reveal the neural control mechanism of sensorimotor system to control the hand-grasping function achievement by regulating the intensity of neuronal synchronization oscillations.

## 1. Introduction

The key to sensorimotor control lies in the interaction between the motor cortex and the muscles involved in the movement (Chen et al., 2018). In the process of movement, the motor cortex sends nerve impulses through the brainstem and spinal cord along the motor nerve pathway to innervate muscle contraction and drive the skeleton to complete the corresponding action. At the same time, the proprioceptive sensations generated by muscle contraction and limb movement are integrated and analyzed along the sensory nerve feedback pathway to the cortex to output decision-making instructions and finally complete the movement accurately. The motor nervous system transmits motor control information through neural oscillations, causing synchronous oscillatory activity in motor units. This synchronous oscillatory activity reflects the functional connection between the cortex and muscles, i.e., corticomuscular coupling (CMC) connection (Bourguignon et al., 2019). The electroencephalogram (EEG) of the motor cortex is a general reflection of the electrophysiological activity of brain nerve cells on the surface of the cerebral cortex or scalp, and the surface electromyography (EMG) of the muscles involved in movement is the temporal and spatial superposition of action potentials of motor units in numerous muscle fibers, both of which reflect motor control information and functional response information of the muscle to the brain's control intention, respectively. Therefore, the coupled connections between cortex and muscle can be measured by simultaneous coupling analysis of EEG-EMG signals.

Human working life is inseparable from the dexterous movement of hands, among which precise grasping is the most important form of hand function, which is the basis for various precise and complex operations. Research on the neural control and perceptual feedback mechanisms of hand movement functions is of great value in both basic research and clinical diagnosis of diseases. Sensory-motor dysfunction of the upper limbs not under the autonomic control of the brain due to neurological impairment is more common, and hand-grasping function is an important index for clinical rehabilitation evaluation (Bao et al., 2021). Therefore, the coupling connection between the sensorimotor cortex and muscles during the execution of precise hand-grasping can be used as a basis for exploring the mechanism of sensorimotor control and clinical rehabilitation evaluation. Since the discovery of the correlation between EEG and EMG signals during exercise in 1995 (Conway et al., 1995), researchers have successively conducted studies on the relationship between EEG-EMG coherence and locomotion paradigm, analyzing the change characteristics and synchronization patterns of scalp EEG signals and EMG signals for hand movements (Johnson and Shinohara, 2012; Clark et al., 2013). Zhang et al. (2017) based on different grip CMC analysis, confirmed that CMC was mainly reflected in the beta and gamma bands during static grip output, where the coupling intensity in the EEG → EMG direction was higher than that in the EMG → EEG direction. Witte et al. (2007) found an increased frequency of synchronous oscillations in the smaller steady-state force output state of the hand. Some studies further suggested that gamma band CMC was related to participants' attention during task performance, and that enhanced task attention promotes neuronal gamma band activity (Li et al., 2020). Also, it has been suggested that gamma band may occur during finger movement preparation and motor performance control (Tun et al., 2021).

The aforementioned study showed that synchronized oscillations in different frequency bands between cortex and muscles reflect different functional coupling relationships. The coupling relationships in different frequency bands are related to hand force output and task attention. The above EEG-EMG synchronization characteristics based on the traditional coupling analysis methods can not reflect the functional interaction information transfer between the cerebral cortex and the corresponding muscles, while Granger causality analysis was applied to the EEG-EMG synchronization studies because of its ability to describe the causal information transfer pattern, and found a bidirectional coupling between EEG and EMG (She et al., 2019). Modeling has a wide range of applications in the biomedical field (Gu et al., 2020; Li et al., 2022a). Granger causality was originally developed in the context of linear autoregressive models with exogenous inputs (ARX) and has been extensively studied in the time and frequency domains, respectively (Zhao et al., 2013; He et al., 2014). However, due to the inherent non-stationary and non-linearity of EEG and EMG signals, traditional methods of Granger causality analysis may not be sufficient to effectively reveal the underlying non-stationary and non-linearity. Therefore, this paper combines the time-varying nonlinear autoregressive with exogenous input model (TV-NARX) with the Granger causality analysis approach. At the same time, considering that it is more practical to study the variation pattern of EEG-EMG synchronization in the time-frequency domain, the frequency resolution cannot be too low, otherwise the detailed information will be lost. Based on the high frequency resolution of wavelet packet transformation, this paper proposes the wavelet packet transform time-frequency Granger causality (WPT-TF-GC) method to better characterize the time-frequency domain of EEG-EMG synchronous coupling, detect nonlinear dynamic effects and track the changes of these effects over time, explore the motor control and feedback response mechanisms of cortical muscles under different hand-grasping movements, and provide a neurophysiological basis for the application of neuromuscular synchronous coupling connections in basic research and clinical rehabilitation evaluation.

## 2. Methods

### 2.1. Data and pre-processing

**Figure 1:**
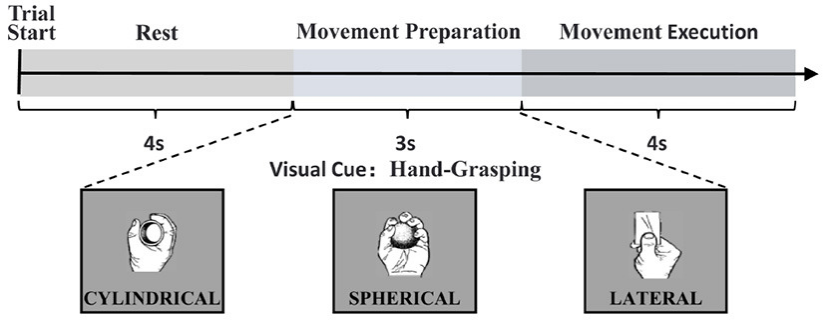
Experimental paradigm in a single trial and the representation of visual cues according to each task.

The data for this study were obtained from the open access database of Korea University (Jeong et al., 2020), which collected 60 EEG electrodes to record cortical responses to upper limb movements according to the 10–20 international configuration, and 7 Ag/AgCl electrodes to record EMG using a sampling frequency of 2,500 Hz, recording experimental data for a total of 11 different upper limb movements in 25 participants. When the experiment started, visual instructions were provided on a monitor. Participants stared at the visual instructions for 4 s at rest. After the rest, the visual cue for the task was given for 3 s and the participants performed the corresponding task within 4 s. And the task was performed a total of 50 times. The experimental flow is shown in Figure 1.

Coherence between EMG and EMG has been found to exist in primates performing fine hand tasks (Beck et al., 2021). The hand-grasping function is mainly associated with the finger extensors and finger flexors (Mangold et al., 2005), and the focus of this study is on the corticomuscular interaction of the human sensorimotor system. Therefore, EEG signals from C3 channel of the main sensorimotor region and surface EMG signals from finger extensors of three designated grasp movements by holding the objects, namely, card, ball, and cup with different degrees of fineness were selected for the study in 15 participants.

The EEG and EMG signals were preprocessed using MATLAB programming software based on signal processing and its toolbox EEGLAB (Iversen and Makeig, 2019), and the process was referred to relevant studies to select the appropriate parameters (Bao et al., 2018; Scba et al., 2021). The data preprocessing steps are described below. First, EEG signals were common average referenced. The EEG-EMG signals were 1–60 Hz bandpass filtered, down sampled to 250 Hz, separated into 7 s segments (3 s before and 4 s after the start of the movement), and splitting the data into two analysis periods: the first 3 s defined as the movement preparation period and the last 4 s defined as the movement execution period, and baseline corrected. Independent Component Analysis (ICA) was then applied to extract EEG source activity, and the sources of independent components were automatically labeled using IClabel to remove components associated with eye movement, muscle, and heartbeat artifacts. ICA is a widely used pre-processing technique that decomposes EEG signals into independent components; IClabel is a classifier that automatically labels the independent components of EEG signals, and this two can be used together to separate and remove noise sources (Pion-Tonachini et al., 2019). In addition, line noise is removed using the EEGLAB CleanLine plug-in to reduce the external electrical noise interference in the original signal. The segmented data is superimposed and averaged to further remove spontaneous background noise. In the original experimental data set, there were scale differences in the amplitudes of the EEG and EMG signals. To avoid pathological problems in the relevant calculation steps, the data were normalized so that their amplitudes had similar scales (Gu et al., 2020).

### 2.2. WPT-TF-GC

The synchronization oscillations between cortical and muscles at different times and frequency bands reflect different functional coupling relationships, so it is more practical to study the variation patterns of EEG-EMG synchronization in the time-frequency domain than in the time or frequency domain alone. Wavelet transform can provide both time domain and frequency domain information of the signal, and is an effective method to analyze EEG and EMG signals. However, wavelet transform only further decomposes the low frequency part of the signal, and does not continue to decompose the high frequency part, i.e., the detailed part of the signal, which has poor frequency resolution in the high frequency band and poor time resolution in the low frequency period. Therefore, the wavelet packet analysis method with better time-frequency resolution is selected in this study to extract the EEG and EMG signals in each time-frequency band. The wavelet packet transform is an extension of wavelet transform, which can effectively decompose the high frequency part of wavelet transform without subdivision and select the corresponding frequency band adaptively according to the characteristics of the analyzed signal, to improve the time-frequency resolution (Chinara et al., 2021). The wavelet packet transform with arbitrary multi-scale feature avoids the defects of wavelet decomposition with fixed time and frequency, which provides a great choice for time-frequency analysis. So, it can better reflect the nature and characteristics of signals.

#### 2.2.1. WPT

**Figure 2:**
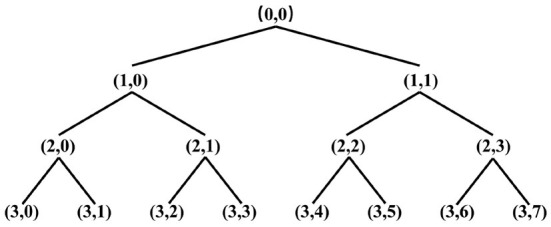
Three-layer Wavelet packet decomposition structure.

The wavelet packet transform yields a binomial tree structure, and the binomial tree nodes are noted as (*j, i*), *j* is the number of decomposition layers, *i* is the number of nodes in the layer. After wavelet packet transform, the original signal is divided into several frequency bands, and the frequency resolution of each band is *fs*/(2^*j*+1^) Hz, *fs* is the sampling frequency. The three-layer decomposition structure of the wavelet packet is shown in Figure 2, (0,0) indicates the original signal, (*j, i*) denotes the wavelet packet coefficient of the corresponding node *d*_*j, i*_, where *j* = 0, 1, 2, 3, *i* = 0, 1, 2, ..., 7. Let *S*_3, *i*_ be the reconstruction of *d*_3, *i*_, then the original signal can be expressed as:


(1) $$\begin{array}{l} S=S_{3,0}+S_{3,1}+S_{3,2}+S_{3,3}+S_{3,4}+S_{3,5}+S_{3,6}+S_{3,7} \end{array}$$

In this study, feature extraction is performed using a seven-layer Coiflets wavelet packet that segments EEG and EMG signals and extracts their time-frequency domain coefficients up to the seventh layer of the wavelet packet transform (Venkata Phanikrishna et al., 2021), which yields the amplitude features *w*(*f, t*) of EMG and EEG at a specific frequency *f* at time point *t*. When the frequency resolution is 1 Hz, neither the detailed information of EEG and EMG signal changes with frequency nor the frequency overlap phenomenon will be lost (Zhang et al., 2017). Therefore, in this study, the frequency resolution is set to 1 Hz, and the EEG and EMG signals with center frequency *f* = 1, 2, ..., 50 Hz are extracted, respectively.

#### 2.2.2. GC

A classical method to detect causal influence between two coupled signals is the Granger causality test. Suppose *X* and *Y* are two signals whose time observations are denoted by *x*(*t*), *y*(*t*), where *t* = 1, 2, ..., *N*. In order to calculate the Granger causality from *X* to *Y*, an unbiased model must be built in advance, which defines the relationship between the output *Y* and the past information *Y*^−^ and the input *X* and the past information *X*^−^, denoted as: *Y* = *f*(*Y*^−^, *X*^−^). Based on the sampled data, the parameters *f*(*Y*^−^, *X*^−^) in the model are estimated, and then predictions of *Y* are generated based on *Y*^−^ only and based on *Y*^−^ and *X*^−^, respectively. In both cases, the model prediction error variance *var*(*Y*|*Y*^−^), *var*(*Y*|*Y*^−^, *X*^−^), is used to represent the accuracy of the prediction. Then the Granger causality of *X* to *Y*, defined as:


(2) $$\begin{array}{l} G_{{\;}_{X\to Y}}=\ln\;\frac{\mathrm{var}\left(Y|Y^{-}\right)}{\mathrm{var}\left(Y|Y^{-},X^{-}\right)} \end{array}$$

And vice versa for the causality from *Y* to *X*. An advantage of this approach is the ability to detect bidirectional causality, since causality from *Y* to *X* and from *X* to *Y* is computed independently, and the method can be used for both linear and nonlinear systems if the model structure is properly chosen.

#### 2.2.3. NARX and TV-NARA model

The NARX model was first proposed by Billings and Leontatis and can describe a wide range of nonlinear dynamic systems (Billings, 2013). The input-output relationship of a nonlinear system can be expressed using a polynomial NARX model with *y*(*f*), *u*(*f*) denoting a segment of EEG and EMG time series at a specific frequency *f* after preprocessing, respectively, with a univariate NAR model as:


(3) $$\begin{array}{l} y\left(f,t\right)=\overset{p}{\underset{k=1}{\sum }}a_{1,k}y\left(f,t-k\right)+\overset{p}{\underset{k=1}{\sum }}\overset{p}{\underset{j=1}{\sum }}a_{3,k,j}y\left(f,t-k\right)y\left(f,t-j\right)\\ \;+e_{y}\left(f,t\right) \end{array}$$

(4) $$\begin{array}{l} \mathrm{var}\left(Y|Y^{-}\right)=\overset{N}{\underset{t=1}{\sum }}{\left(\hat{y}\left(f,t\right)-y\left(f,t\right)\right)}^{2}/N \end{array}$$

(5) $$\begin{array}{l} x\left(f,t\right)=\overset{p}{\underset{k=1}{\sum }}b_{1,k}x\left(f,t-k\right)+\overset{p}{\underset{k=1}{\sum }}\overset{p}{\underset{j=1}{\sum }}b_{3,k,j}x\left(f,t-k\right)x\left(f,t-j\right)\\ \;+e_{x}\left(f,t\right) \end{array}$$

(6) $$\begin{array}{l} \mathrm{var}\left(X|X^{-}\right)=\overset{N}{\underset{t=1}{\sum }}{\left(\hat{x}\left(f,t\right)-x\left(f,t\right)\right)}^{2}/N \end{array}$$

which *a*_1,*k*_, *a*_3,*k,j*_, *b*_1,*k*_, *b*_3,*k,j*_ is the model coefficient, *p* is the number of model orders, and *e*_*y*_(*f, t*), *e*_*x*_(*f, t*) is the model prediction error, *N* is the number of samples, and *var*(*Y*|*Y*^−^), *var*(*X*|*X*^−^) represent the variance of the univariate NAR models for the series *y*(*f*), *u*(*f*), respectively. Considering the EEG and EMG signals together, the bivariate NARX model can be expressed as:


(7) $$\begin{array}{l} \;y\left(f,t\right)=\overset{p}{\underset{k=1}{\sum }}a_{1,k}y\left(f,t-k\right)+\overset{q}{\underset{k=1}{\sum }}a_{2,k}x\left(f,t-k\right)\\ +\overset{p}{\underset{k=1}{\sum }}\overset{p}{\underset{j=1}{\sum }}a_{3,k,j}y\left(f,t-k\right)y\left(f,t-j\right)\\ +\overset{q}{\underset{k=1}{\sum }}\overset{q}{\underset{j=1}{\sum }}a_{4,k,j}x\left(f,t-k\right)x\left(f,t-j\right)\\ +\overset{p}{\underset{k=1}{\sum }}\overset{q}{\underset{j=1}{\sum }}a_{5,k,j}y\left(f,t-k\right)x\left(f,t-j\right)+e_{y}\left(f,t\right) \end{array}$$

(8) $$\begin{array}{l} \mathrm{var}\left(Y|Y^{-},X^{-}\right)=\overset{N}{\underset{t=1}{\sum }}{\left(\hat{y}\left(f,t\right)-y\left(f,t\right)\right)}^{2}/N \end{array}$$

(9) $$\begin{array}{l} x\left(f,t\right)=\overset{p}{\underset{k=1}{\sum }}b_{1,k}x\left(f,t-k\right)+\overset{q}{\underset{k=1}{\sum }}b_{2,k}y\left(f,t-k\right)\\ +\overset{p}{\underset{k=1}{\sum }}\overset{p}{\underset{j=1}{\sum }}b_{3,k,j}x\left(f,t-k\right)x\left(f,t-j\right)+\\ \overset{q}{\underset{k=1}{\sum }}\overset{q}{\underset{j=1}{\sum }}b_{4,k,j}y\left(f,t-k\right)y\left(f,t-j\right)\\ +\overset{p}{\underset{k=1}{\sum }}\overset{q}{\underset{j=1}{\sum }}b_{5,k,j}x\left(f,t-k\right)y\left(f,t-j\right)+e_{x}\left(f,t\right) \end{array}$$

(10) $$\begin{array}{l} \mathrm{var}\left(X|X^{-},Y^{-}\right)=\overset{N}{\underset{t=1}{\sum }}{\left(\hat{x}\left(f,t\right)-x\left(f,t\right)\right)}^{2}/N \end{array}$$

which *a*_1, *k*_, ..., *a*_5, *k, j*_, *b*_1, *k*_, ..., *b*_5, *k, j*_ is the model coefficient, *q* is the number of model orders. *var*(*Y*|*Y*^−^, *X*^−^), *var*(*X*|*X*^−^, *Y*^−^) represent the variance of the univariate NARX models for the series *y*(*f*), *u*(*f*), respectively. Then the nonlinear causal impact between *X* and *Y* can be measured by the following indicators.


(11) $$\begin{array}{l} G_{{\;}_{X\to Y}}=\ln\;\frac{\mathrm{var}\left(Y|Y^{-}\right)}{\mathrm{var}\left(Y|Y^{-},X^{-}\right)} \end{array}$$

(12) $$\begin{array}{l} G_{{\;}_{Y\to X}}=\ln\;\frac{\mathrm{var}\left(X|X^{-}\right)}{\mathrm{var}\left(X|X^{-},Y^{-}\right)} \end{array}$$

The initial full regression set of NAR, NARX may be highly redundant, and some regression quantities or model terms have little impact on the predictive power of the model and can be removed from the initial regression equation, and this elimination of redundant regression quantities usually improves the model performance. For most nonlinear dynamical system identification problems, only a relatively small number of model terms are usually required in the final regression model (Gu et al., 2020). Therefore, an efficient model term detection algorithm is needed to detect and select the most important regression quantities. In this paper, the forward regression least squares method is used to be able to detect the model structure that explains the key features of the data. The key to the model structure detection problem is how to find a subset *D*_*n*_ = {φ_*l*_1__, φ_*l*_2__, …, φ_*l*_*n*__}({*l*_1_, *l*_2_, …, *l*_*n*_} ∈ {1, 2, …, *M*}) from the initial set of candidate model items *D* = {φ_1_, φ_2_, …, φ_*M*_} (*M* is the number of model terms) such that y can be approximated by a linear combination: *y* = θ_*l*_1__φ_*l*_1__+θ__*l*_2___φ_*l*_2__+…+θ_*l*_*n*__φ_*l*_*n*__ +*e*, which e is model prediction error.

Step 1: Calculate the creation of all *M* model terms and calculate the EER set:


(13) $$\begin{array}{l} \;\psi \;=\left[\epsilon_{1},\epsilon_{2},\epsilon_{3},\cdots \;,\epsilon_{M}\right] \end{array}$$

(14) $$\begin{array}{l} \epsilon_{m}={\left(y^{T}\varphi_{m}\right)}^{2}/\left(y^{T}y\right)\left(\varphi_{m}^{T}\varphi_{m}\right) \end{array}$$

(15) $$\begin{array}{l} l_{1}=arg\underset{1\leq m\leq M}{\max}\left\{\epsilon_{m}\right\} \end{array}$$

The model term with the largest value of ϵ_*m*_ is selected as the first valid model term with index *l*_1_ and is denoted as φ_*l*_1__. Then φ_*l*_1__ is the first valid model term selected and the first associated orthogonal vector is defined as *q*_1_ = φ_*l*_1__. When a valid model term is selected, it should be deleted from the set of candidate model terms, and then the set of candidate model items is reduced to *M*−1.

Step *s*(*s* ≤ 2): the remaining set of *M*−*s*+1 candidate model terms need to be transformed into a new set of orthogonalized vectors by Gramm-Schmidt (GS) transformation. The GS transformation can be implemented by the following equation:


(16) $$\begin{array}{l} q_{j}^{s}=\varphi_{j}-\overset{s-1}{\underset{r=1}{\sum }}\frac{{\left(\varphi_{j}\right)}^{T}q_{r}}{{\left(q_{r}\right)}^{T}q_{r}}q_{r} \end{array}$$

where *q*_*r*_(*r* = 1, 2, …, *s*−1) is the orthogonal vector, φ_*j*_(*j* = 1, 2, …, *M*−*s*+1) is the unselected model terms, and $q_{j}^{s}$ is the new orthogonal vectorization. The ERR matrix for step *s* is then computed using $\left[q_{1}^{s},q_{2}^{s},\ldots ,q_{M-s+1}^{s}\right]$:


(17) $$\begin{array}{l} \;\psi \;=\left[\epsilon_{1},\epsilon_{2},\epsilon_{3},\cdots \;,\epsilon_{M-s+1}\right] \end{array}$$

(18) $$\begin{array}{l} \epsilon_{m}={\left(y^{T}\varphi_{m}\right)}^{2}/\left(y^{T}y\right)\left(\varphi_{m}^{T}\varphi_{m}\right) \end{array}$$

(19) $$\begin{array}{l} l_{s}=arg\underset{1\leq m\leq M-s+1}{\max}\left\{\epsilon_{m}\right\} \end{array}$$

φ_*l*_*s*__ is selected as the *s*-th valid model term, and the *s*-th associated orthogonal vector is defined as *q*_*s*_ = φ_*l*_*s*__. Therefore, the subsets [φ_*l*_1__, φ_*l*_2__, ..., φ_*l*_*n*__] are selected step by step. Finally, the model parameter vector θ = [θ_*l*_1__, θ_*l*_2__, ..., θ_*l*_*n*__] is estimated by the triangular formula $A\theta \;=\;{\left(y\right)}^{T}q_{j}/{\left(q_{j}\right)}^{T}q_{j}\left(j=1,2,\ldots ,n\right)$, where *A* is the unit-on-unit triangular matrix and *n* is the number of model terms finally selected. If the interaction between two signals is time-varying rather than stationary, the complexity of the model will increase significantly. When dealing with time-varying problems, the assumption of “short -time invariance” is often adopted, i.e., the time-varying parameters are treated as constants for a very short period (Chen et al., 2020). Under this assumption, given a suitable short time interval Δ*t*, for any time step *t*_*n*_, within the time interval [*t*_*n*_-0.5Δ*t*, *t*_*n*_+0.5Δ*t*], Equation (20) can be treated as a time-invariant system to fit the full TV-NARX model by gradually sliding the window in the data.


(20) $$\begin{array}{l} y\left(f,t\right)=\overset{p}{\underset{k=1}{\sum }}a_{1,k}\left(t\right)y\left(f,t-k\right)+\overset{q}{\underset{k=1}{\sum }}a_{2,k}\left(t\right)x\left(f,t-k\right)\\ +\overset{p}{\underset{k=1}{\sum }}\overset{p}{\underset{j=1}{\sum }}a_{3,k,j}\left(t\right)y\left(f,t-k\right)y\left(f,t-j\right)+\\ \overset{q}{\underset{k=1}{\sum }}\overset{q}{\underset{j=1}{\sum }}a_{4,k,j}\left(t\right)x\left(f,t-k\right)x\left(f,t-j\right)\\ +\overset{p}{\underset{k=1}{\sum }}\overset{q}{\underset{j=1}{\sum }}a_{5,k,j}\left(t\right)y\left(f,t-k\right)x\left(f,t-j\right)+e_{y}\left(f,t\right) \end{array}$$

The time-varying non-linear causal effect of *X* to *Y* can then be measured by the following equation:


(21) $$\begin{array}{l} G_{{\;}_{X\to Y}}=\ln\;\frac{\mathrm{var}\left(Y|Y^{-}\right)\left(t\right)}{\mathrm{var}\left(Y|Y^{-},X^{-}\right)\left(t\right)} \end{array}$$

### 2.3. Statistical analysis

In order to better compare the statistical differences in GC among frequency bands and time periods for different hand-grasping movements, statistical analyses of GC across frequency bands and time periods for the three grasping actions in the directions EEG→ EMG and EMG→ EEG were performed using SPSS data analysis software (Field, 2013) and MATLAB programming software to test the effects of intra-group (different frequency bands) and inter-group factors (different hand-grasping movements) on the observed variables. Before conducting statistical analysis, GC should be performed to meet the normal distribution and variance chi-square, if it meets the normal distribution and variance chi-square, one-way ANOVA is used; if it does not meet the normal distribution then multiple independent samples Kruskal-Wallis test is selected.

## 3. Results

**Figure 3:**
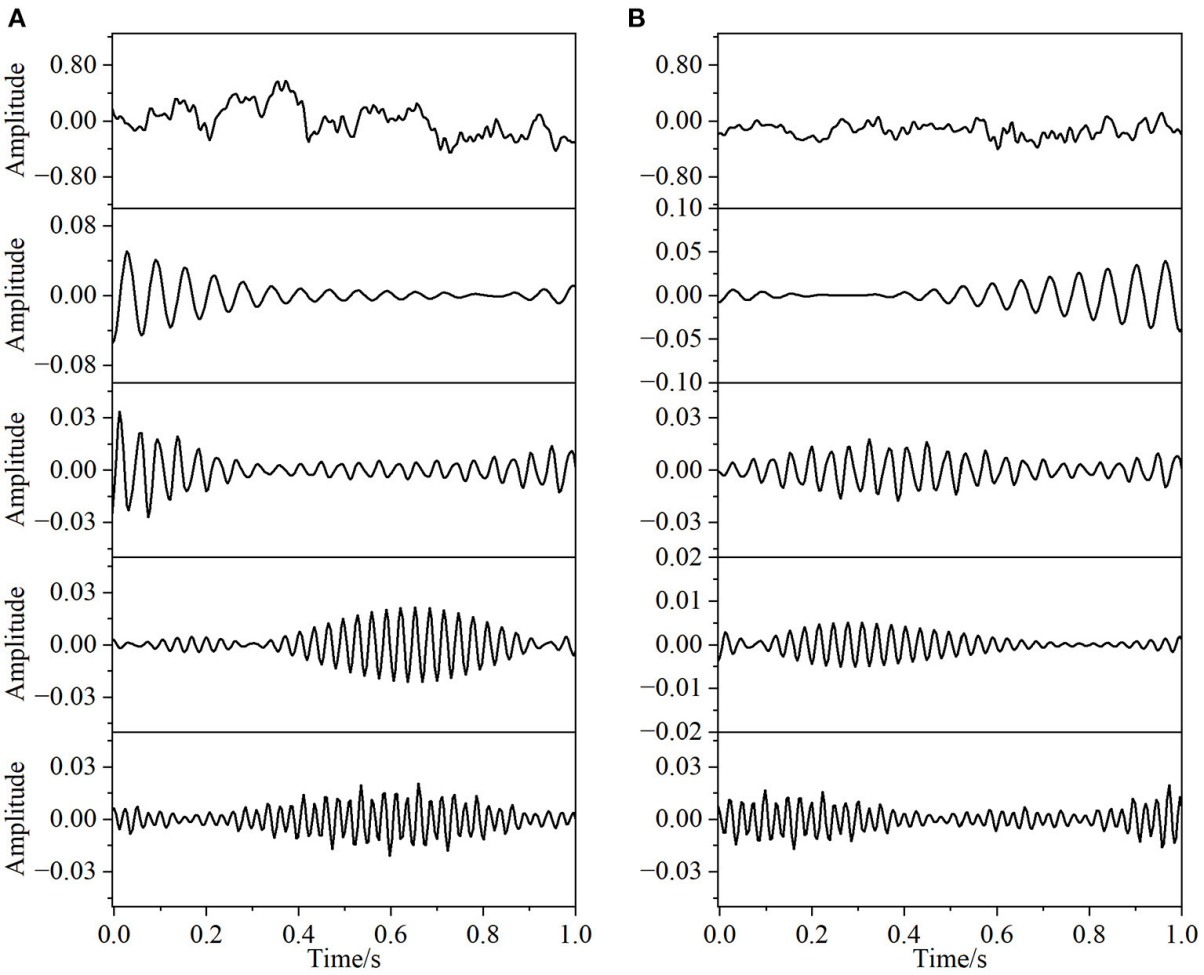
Coiflets wavelet packet transform of the signal (from top to bottom, the normalized signal after pre-processing, extracted after wavelet packet transform for 16, 24, 32, and 40 Hz bands, respectively). **(A)** EEG, **(B)** EMG.

**Figure 4:**
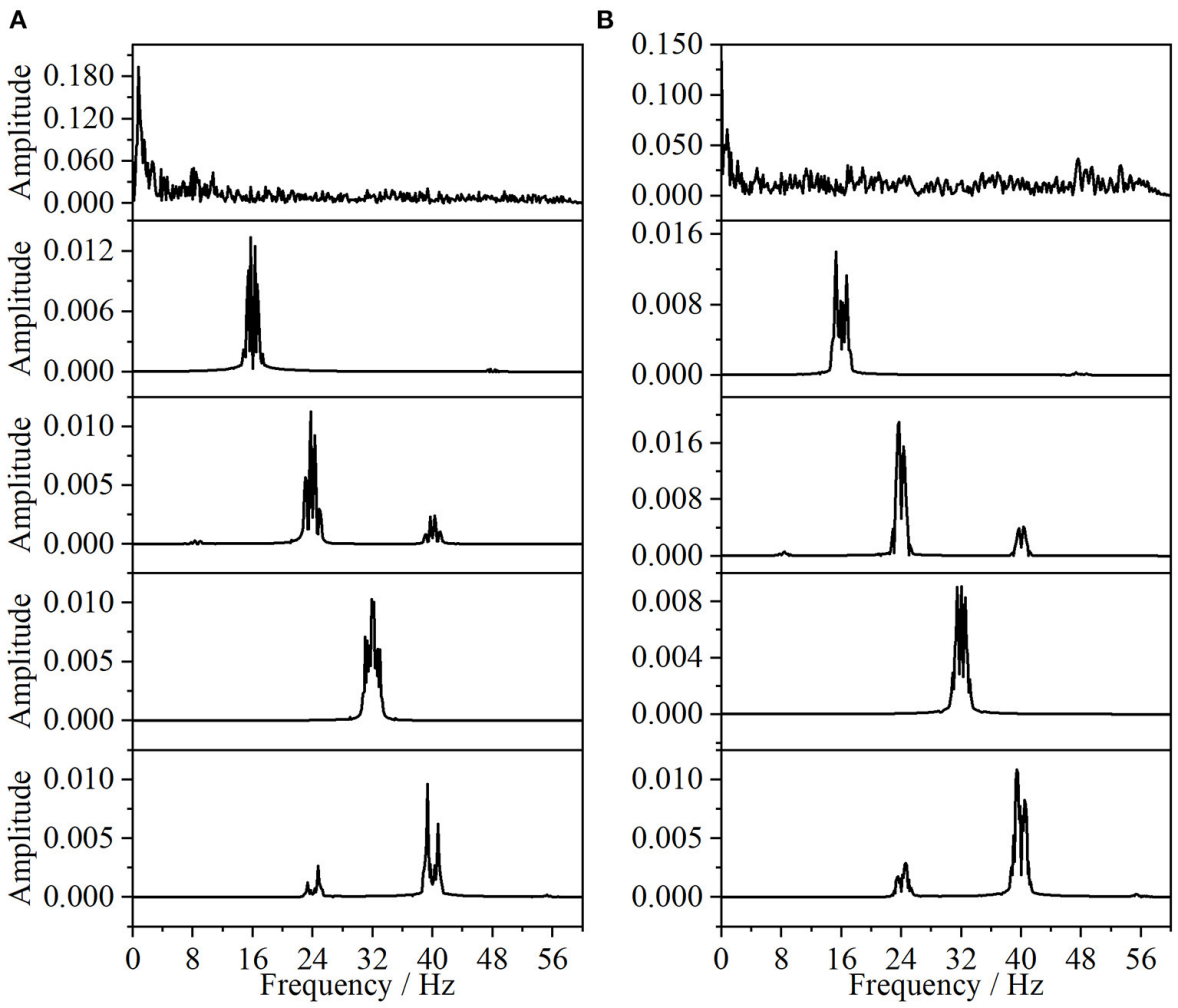
Spectrum of wavelet packet decomposition signal (from top to bottom, the normalized signal after pre-processing, extracted after wavelet packet transform for 16, 24, 32, and 40 Hz bands, respectively). **(A)** EEG, **(B)** EMG.

In this paper, the EEG and EMG signals of three hand-grasping movements in 15 healthy participants in Jeong et al. (2020) were selected and preprocessed according to the method in Section 1.2 to obtain data with a duration of 7 s under each movement. According to the method in Section 2.1.1 of this paper, Coiflets wavelet packet decomposition is applied to extract the EEG and EMG signals in each frequency band from 1 to 50 Hz. Figure 3 shows the normalized EEG and EMG signals after pre-processing and the extracted EEG and EMG signals at 16, 24, 32, and 40 Hz after wavelet packet transformation. The mean square error between the combined signal of each wavelet packet transform and the original signal is less than 1 × 10^−20^, which shows that the wavelet packet transform does not lose the information of the original signal and confirms the effectiveness of the wavelet packet decomposition. Meanwhile, the spectrum plot with the corresponding frequency of Figure 3 is given in Figure 4, and it can be seen that after Coiflets wavelet packet transform, EEG and EMG signals can be well extracted in each band. The WPT-TF-GC relationship between EEG and EMG signals in each frequency band was calculated according to the method in Section 2.1. To explore the bidirectional causality between EEG and EMG during the execution of different hand-grasping movements in healthy participants, the results of GC analysis of 15 participants were used to illustrate. To quantitatively compare the differences of EEG-EMG causality among participants in different grasping movements, different frequency bands and different time periods, the GC during the grasping of cards, balls, and cups in 15 participants were analyzed.

### 3.1. General trend analysis of EEG-EMG GC in different frequency bands

**Figure 5:**
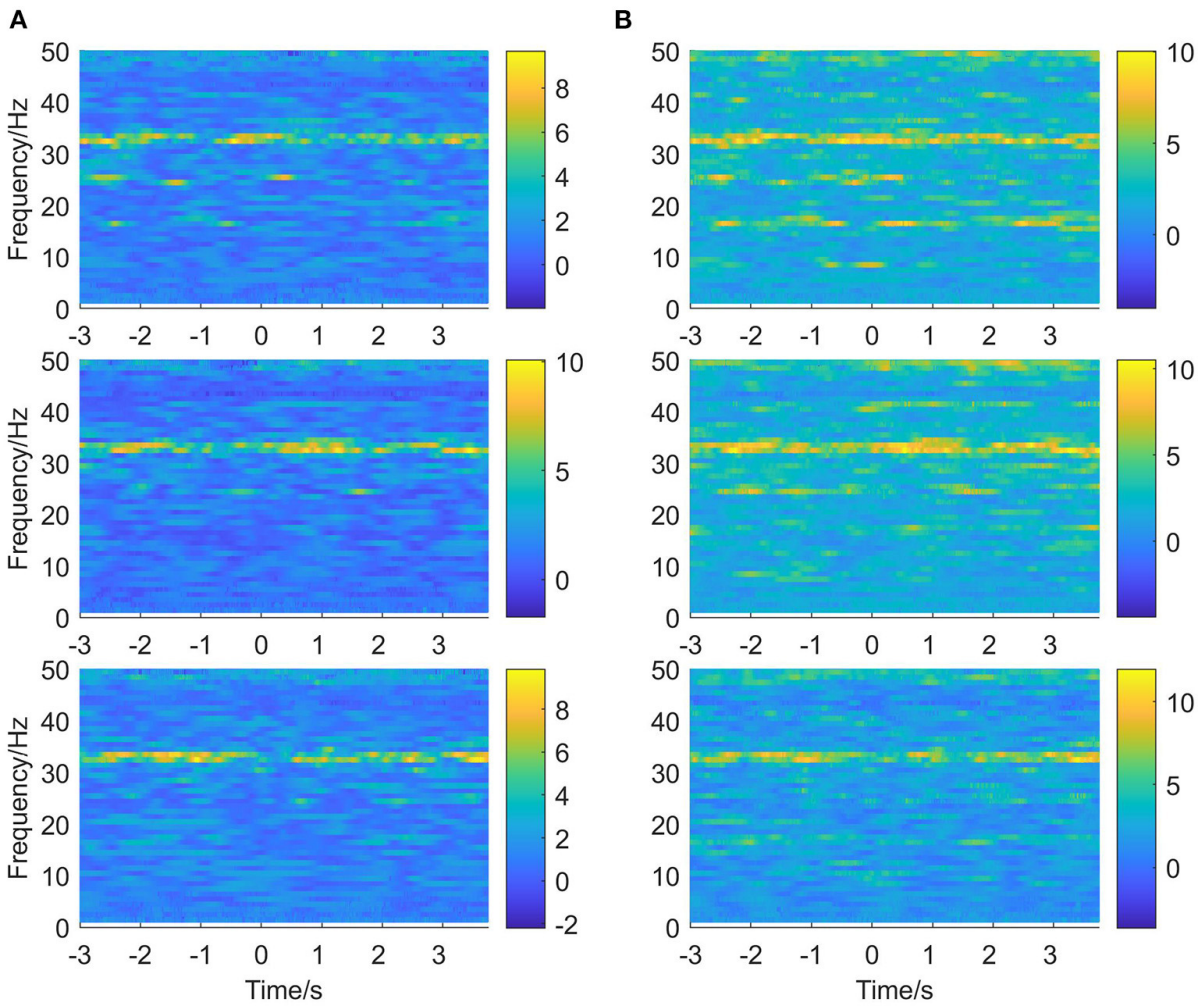
Time-frequency plots of EEG-EMG GC in the two directions of EEG→ EMG and EMG→ EEG under different hand-grasping movements (from top to bottom, card, ball, and cup hand-grasping movement, respectively). **(A)** EMG→ EEG, **(B)** EEG→ EMG.

The EEG signals and EMG signals of 15 participants under different grasping movements were calculated separately according to the methods in Sections 2.1 and 2.2 for bidirectional WPT-TF-GC: *G*_*EEG*→*EMG*_ and *G*_*EMG*→*EEG*_. Figure 5 shows the GC time-frequency plots of participants in the two directions of EEG→ EMG and EMG→ EEG under three different hand grasping movements. The horizontal coordinate is time and the vertical coordinate is frequency, which can clearly show the variation pattern of the EEG-EMG synchronous coupling relationship in the time-frequency domain, and the GC intensity in the EEG→ EMG direction is higher than that in the EMG→ EEG direction. The significant frequency bands of *G*_*EEG*→*EMG*_ are scattered in the alpha band, beta band and gamma band, with the beta band being the most significant and the gamma band the second most significant; while the significant frequency bands of *G*_*EMG*→*EEG*_ are mainly concentrated in the beta band and gamma band. This indicates that in the state of precise hand movements, the corticomuscular functional coupling is bidirectional, where the coupling strength in the EEG→ EMG direction is higher than that in the EMG→ EEG direction. The instructions of movement control were mainly transmitted to muscles through alpha, beta, gamma and caused oscillations of beta, gamma of EMG; while the EEG-EMG coupling caused by sensory feedback from muscles to cortex was significant in mainly in the beta frequency band. The time-frequency plot of one of the participants was given in Figure 5, and the remaining 14 showed similar patterns of changes in the time-frequency domain.

**Figure 6:**
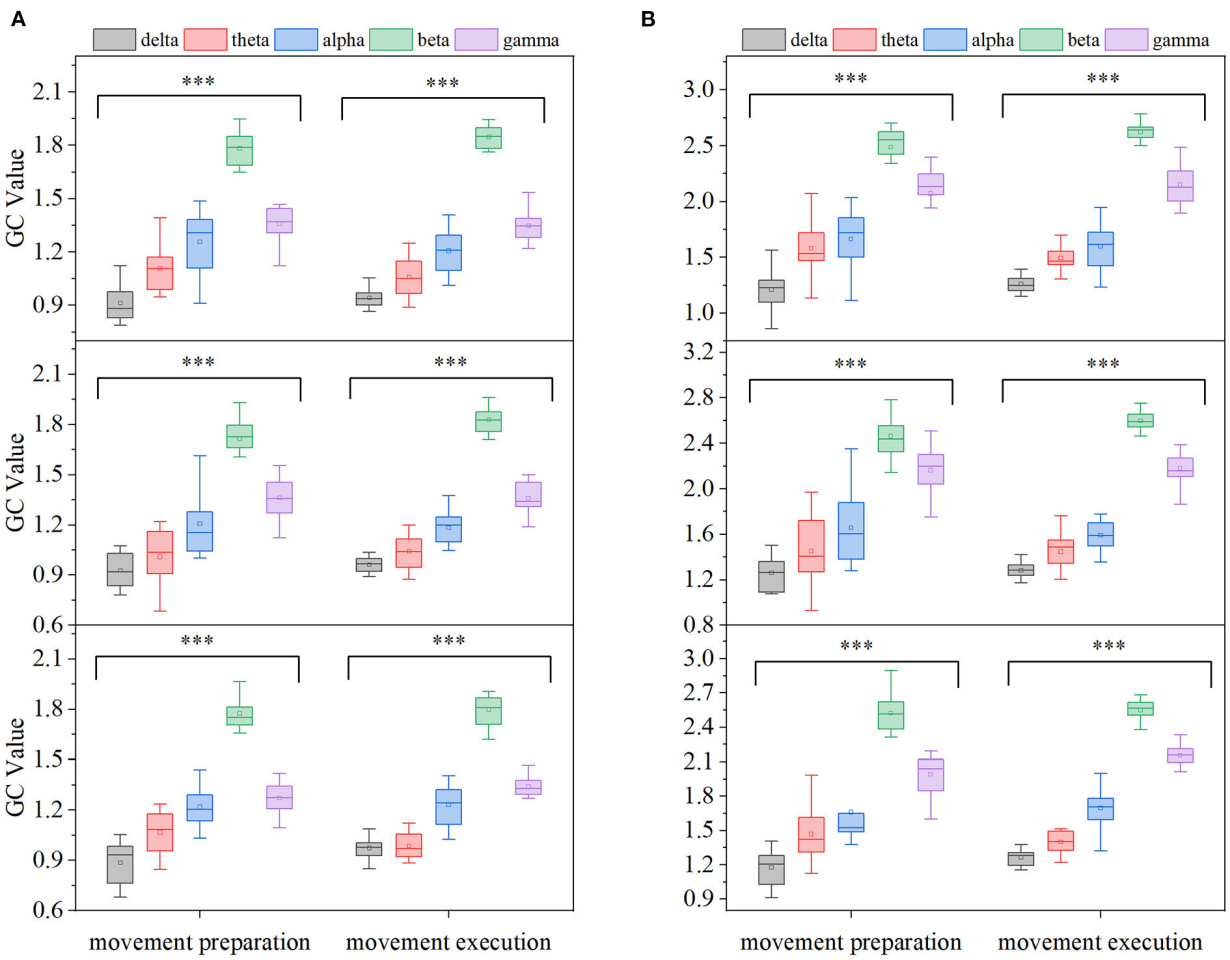
Comparison of GC averages under different frequency bands (from top to bottom, card, ball, and cup hand-grasping movement, respectively, ***p < 0.01). **(A)** EMG→ EEG, **(B)** EEG→ EMG.

In order to more clearly describe the EEG-EMG GC characteristics at different frequency bands and compare the differences in EEG-EMG coupling intensity in each frequency band, the GC averages in five EEG frequency bands [delta (0.5–4 Hz), theta (4–8 Hz), alpha (9–12 Hz), beta (13–35 Hz), gamma (>35 Hz)] were calculated for all participants in the EEG→ EMG and EMG→ EEG directions and during the movement preparation and movement execution periods, respectively, and the results are shown in Figure 6. Comparing on different frequency bands, the GC of beta and gamma bands were significantly higher than that of delta, theta, and alpha bands under three movements and two different time periods. To further test whether the different frequency bands of *G*_*EEG*→*EMG*_ and *G*_*EMG*→*EEG*_ were significant at different movements and different time periods, a one-way ANOVA was performed on the GC data of 15 participants according to the method in Section 2.2. It was found that there were significant differences among the different frequency bands at the three movements and different time periods (*p* < 0.01).

### 3.2. EEG-EMG GC analysis of different grasping movements

**Figure 7:**
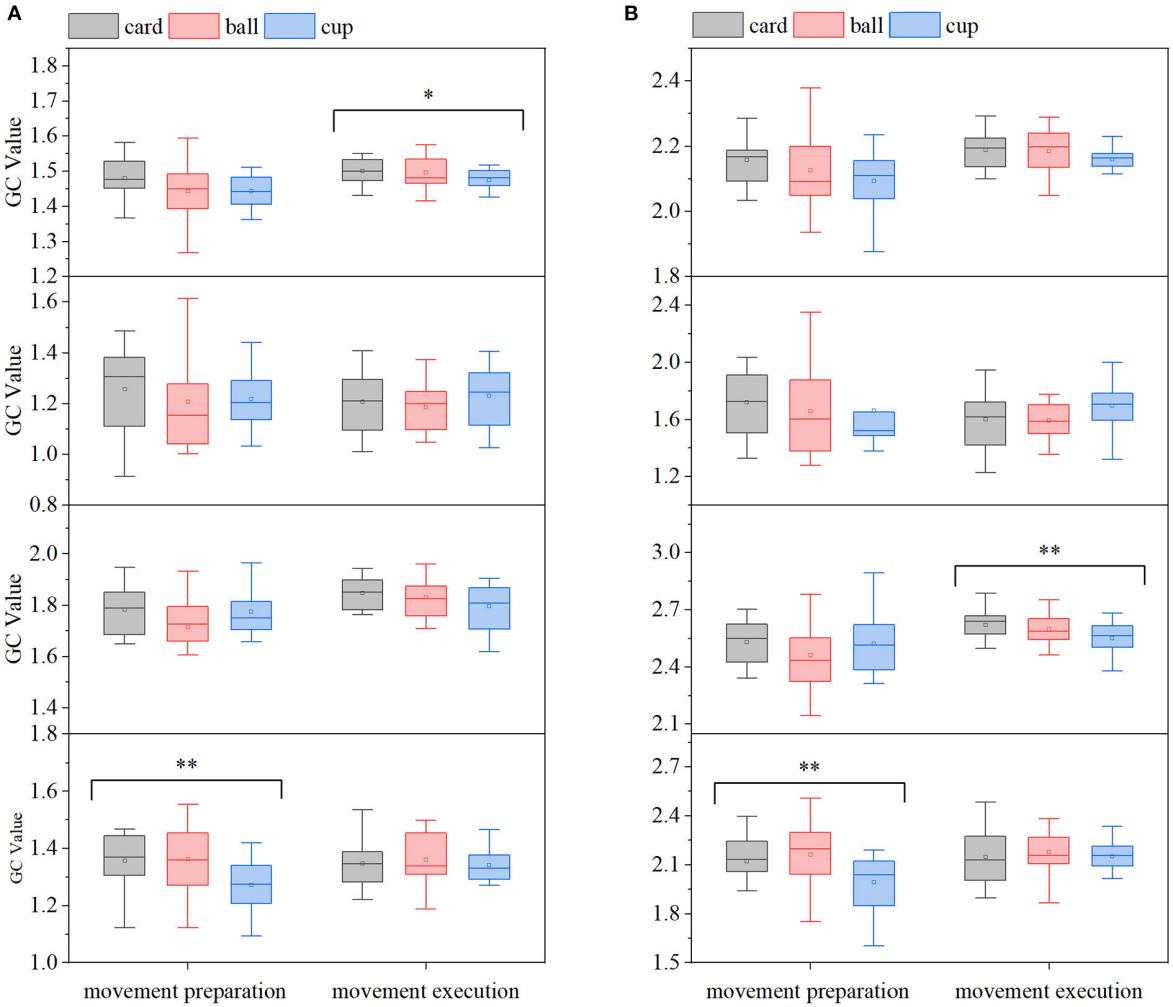
Comparison of GC averages under different hand-grasping movements (from top to bottom, full frequency, alpha, beta, gamma band, *p < 0.1; **p < 0.05). **(A)** EMG→ EEG, **(B)** EEG→ EMG.

In order to analyze the EEG-EMG GC characteristics and compare the differences in EEG-EMG coupling intensity under different movements, the GC averages in the EEG→ EMG and EMG→ EEG directions in the motor preparation period and movements execution period were first calculated at full frequency for all participants, and the results are shown in Figure 7, in which it can be seen that there are differences in *G*_*EMG*→*EEG*_ among the three grasping movements in the movements execution period. To further examine in which frequency band this difference in EEG-EMG coupling intensity was found specifically, and as shown in Figure 5, the *G*_*EEG*→*EMG*_ and *G*_*EMG*→*EEG*_ of all participants under the three grasping movements were mainly reflected in the alpha, beta, and gamma frequency bands. Therefore, the mean values of GC in the alpha, beta, and gamma frequency bands were calculated for all participants in the two directions of EEG→ EMG and EMG→ EEG during the movement preparation and movement execution periods, as shown in Figure 7. In the gamma band, differences in *G*_*EMG*→*EEG*_ and *G*_*EEG*→*EMG*_ were observed during the movements preparation period, while in the beta band, differences in *G*_*EEG*→*EMG*_ were observed among the movements in the execution period. Also, in order to confirm the significance of the above results, the statistical significant analysis of the GC data of 15 participants was performed according to the method in Section 2.2, and it was found that there were significant differences among the different movements in the full frequency of the movement execution period (*p* < 0.1), the beta frequency band of the movement execution period (*p* < 0.05) and the gamma frequency band of the movement preparation period (*p* < 0.05).

## 4. Discussion

At present, there are not many studies on the EEG-EMG synchronization characteristics under different grasping movements of the hand, and most of them are based on the traditional coherence analysis methods, which cannot give information on the directionality of EEG-EMG coupling. Also, the classical Granger causality based on linear autoregressive models cannot give information on the nonlinear and time-varying aspects of EEG-EMG coupling. In this paper, the WPT-TF-GC algorithm is proposed to explore the differences of nonlinear time-varying characteristics of EEG-EMG synchronization under hand-grasping movements in two dimensions of the time-frequency domain. Compared with the conventional GC, WPT-TF-GC has better time-frequency resolution and can comprehensively explain the time-frequency domain characteristics and directional information of nonlinear EEG-EMG synchronization coupling.

The results of this paper confirm the existence of a bidirectional Granger causality between EEG and EMG in healthy participants during the execution of different hand-grasp reflecting the characteristics of a closed-loop cortical-muscle control loop, in which motor commands from the cortex travel down to the muscle through the control efferent pathway accompanied by afferent neural feedback processes from the contracting muscle. Meanwhile, the coupling intensity in the EEG→ EMG direction was higher than that in the EMG→ EEG direction, reflecting the difference in directional synchronous oscillations between sensory feedback and motor control mechanisms, which is consistent with the previous findings (Zhang et al., 2017).

The coupling strength of EEG-EMG varies in different frequency bands because different frequency bands are involved in different functional coupling oscillations during sensorimotor control. In the comparison of different frequency bands, the EEG-EMG coupling was mainly reflected in the alpha, beta, and gamma bands, indicating that it is the alpha, beta, and gamma bands that dominate in hand grasp motor control (Xie et al., 2019). The highest GC values were found in the beta band, because the beta band is mainly associated with cortical-to-muscle control functions during sensorimotor control and is dependent on proprioceptive afferents (Bourguignon et al., 2019). The cortical-muscle functional coupling in the beta band reflects the relatively stable control state of the sensorimotor cortex. In addition, there were significant differences in *G*_*EEG*→*EMG*_ among movements during movement execution, suggesting that the control state of the beta band of sensorimotor cortex for grasping different objects may also differ.

It has been shown that gamma-band oscillations are associated with changes in attention (Honkanen et al., 2015; Li et al., 2020). Enhancement of task attention promotes neuronal gamma band oscillations. During the movement preparation period, there were significant differences in the gamma frequency bands of the three different grasping movements. This suggests that before the start of the movement, depending on the task performed, the cortical sensory control system already starts to deploy different levels of attention in preparation for the start of the movement, resulting in an accelerated frequency of synchronized oscillations of neuromuscular motor neurons. This may be due to the fact that more motor neurons are required for the control of smaller forceful and delicate hand grasping movements, and the rate of recruited neuronal potentials is accelerated. And the intensity of high-frequency synchronous oscillation varies for different degrees of precise hand gripping action. Witte et al. in their study of 16% MVC grip output compared to an increase in EEG-EMG frequency at 4% MVC grip output, i.e., an increase in the associated peak frequency at smaller force outputs (Witte et al., 2007). This suggests that the rate of neuronal potential release is accelerated during the maintenance of very small static force outputs or during the execution of small force hand precise movements. Recent studies have also indicated that cortical activity in the gamma band is closely related to higher cognitive functions (Liu et al., 2022). It can be inferred that synchronized EEG-EMG gamma band oscillations are involved in the sensory-cognitive activity of task-selective attention, reflecting the integration process of motor information processing associated with task attention.

Deep learning is widely used in the biomedical field (Zeng et al., 2021; Li et al., 2022b; Wu et al., 2022), and subsequent applications of deep learning can be considered in this paper.

## 5. Conclusion

In this study, a WPT-TF-GC analysis method was constructed based on the NARX model and Coiflets wavelet packet transform to investigate the differences in coupling strength, frequency band, and information flow characteristics of simultaneous EEG-EMG signal coupling under different hand-grasping movements. The analysis results show that the WPT-TF-GC method has better resolution and can reveal the EEG-EMG synchronous coupling characteristics from two dimensions of the time-frequency domain. The analysis of EEG-EMG synchronous coupling can help explore the control feedback mechanism of human hand-grasping function and can provide a theoretical basis for the basic research of hand function and motor rehabilitation evaluation.

## Data availability statement

The datasets presented in this study can be found in online repositories. The names of the repository/repositories and accession number(s) can be found in the article/supplementary material.

## Author contributions

FZ wrote this manuscript. FZ, YL, and ZS conduced the conception of the study. YL and WS revised the manuscript critically for important intellectual content. All authors reviewed, revised, and finalized the manuscript. All authors contributed to the article and approved the submitted version.

## Funding

This study was supported in part by the National Nature Science Foundation of China under Grant 61773124.

## Conflict of interest

The authors declare that the research was conducted in the absence of any commercial or financial relationships that could be construed as a potential conflict of interest.

## Publisher's note

All claims expressed in this article are solely those of the authors and do not necessarily represent those of their affiliated organizations, or those of the publisher, the editors and the reviewers. Any product that may be evaluated in this article, or claim that may be made by its manufacturer, is not guaranteed or endorsed by the publisher.
